# Supplementary material for: Effects of water and fertilizer coupling on the physiological characteristics and growth of rabbiteye blueberry
Source: PLoS One. 2021 Jul 6;16(7):e0254013. doi: 10.1371/journal.pone.0254013 (PMC8259967; doi:10.1371/journal.pone.0254013)
Supplement: Attachment — Submitted filename: Response to Reviewers.docx [file pone.0254013.s002.docx]

March 1, 2021

**Mohammad Golam Mostofa, PhD**

Academic Editor

PLOS ONE

**Re: Effects of Water and Fertilizer Coupling on the Physiological Characteristics and Growth of Rabbiteye Blueberry (PONE-D-21-00083R1)**

Dear Mohammad Golam Mostofa, PhD

I, on behalf of all co-authors thank you and reviewers for the detailed review and providing us the opportunity to revise and improve the above-cited manuscript. We have carefully revised our manuscript according to the suggestions/comments made by the reviewer (see below point-to-point reply). In addition, We have uploaded the research data as an Supporting information files in accordance with the requirements of your journal.

We hope that the revised manuscript will be accepted for publication in ‘PLOS ONE’.

We wish you to hear from you in due course

Dr. Delu Wang

Corresponding author

**Reviewers 1**

1. The manuscript should be thoroughly revised with native English speaker. In many cases sentences have no logical flow and therefore it is difficult to find author's message.

**Response**：We thoroughly copyedit our manuscript for language usage, spelling, and grammar, and we employed a professional scientific editing service

2. Line 32-36: In abstract author mentioned maximum level of irrigation water and fertilizer application improved photosynthetic capacity in line 32-34. Whereas, in line 34-35 author stated high fertilizer–medium water treatment increased Chlorophyll contents. Again, in line 35-36 they mentioned medium fertilizer–medium water treatment increased Chlorophyll contents, which is very confusing and contradictory with one another. Please correct it.

**Response**: Said lines are revised now for more clarity. Please see Lines 35-37 of the revision.

3. Line 34-36: Please indicate clearly which treatment combination increased leaf-soluble protein.

**Response**: Line 35. The high fertilizer–medium high water treatment increased leaf-soluble protein contents.

4. Line 40-42: Author should be more careful during writing chemical formula. Please check the whole manuscript to fix similar kind of error.

**Response**: All formulas are rechecked and revised to remove errors in full manuscript; see lines 41-44of the revision .

5. Line 60. Please replace hm-2 with international unit throughout the manuscript.

**Response**: hm^-2^ changed to ha^-1^ in whole manuscript.

6. Please clearly indicate plant to plant and line to line distance in this section.

**Response**: mentioned the distance of 1.5 meters within the plants. Please see Line 121 of the revision.

7. Line 129-136: Authors have mentioned that they used 65º c dry sample to measure Chlorophyll content, SOD activity and MDA content using protocol of Mahawar et al. 2018, 2019 and Khator et al. 2020. However, it was found that above mentioned authors used fresh samples to measure these variables, which is contradictory with the sample used by authors and their reference. Give procedure in details.

**Response**: Fristly, I'm very sorry, all the blueberry leaves were fresh. Corrections have been made in revised manuscript regarding chlorophyll contents, SOD and MDA. Please lines 148-156 of the revision.

8. Line 142-150: It is not clear that which parameters authors included under membership value and which parameters included under anti-membership value. As author recommend optimal water and fertilizer rate with the help of membership value therefore it is very important to know which parameters belongs to membership value and which parameters included under anti-membership value. However, author did not mention it clearly in this section. Author can follow Roy et al. (2021) Agricultural Water Management 246, 106712 to make this portion clearer for reader.

**Response**: Thank you reviewers for their comments. I have made changes here. The experiment was arranged using a varied irrigation and fertilizer under completely randomized. Within this design a total of four water doses were used: W1(1.25L, low), W2 (2.5 L, medium), W3 (3.75 L, medium high), W4 (5 L, highest). Furthermore, three N doses as:F1(F1=29g,low), F2(F2 =59g,medium), F3(F3= 118g,highest) were also supplied. With no fertilization and irrigation as the control, there are 13 treatments in total. Treatments using various regimes of W and F doses were selected for this study and presented in Table 1. Each treatment had 3 replications (3 × 13 = 63), and pots were randomly ordered inside the plastic shed. Interactive effects of W × F on integrated growth performance (IGP) of blueberry were obtained by transforming the data related to physiological and growth responses in following Eqs. (2) and (3), and the results are shown in Table 8.

Membership value = (X - Xmin)/(Xmax - Xmin),	(2)

Anti-membership value = 1- (X - Xmin)/(Xmax - Xmin), (3)

In the above equation, X and x represent the coded and the average calculated value of each treatment, respectively; xmin and xmax represent the minimum and maximum value, respectively, obtained from each parameter from different treatments. To calculate IGP of blueberry, we used Eq. (2) for those growth indices that were positively correlated with W, F application. However, Eq. (3) was used for those growth indices that were negatively correlated with W,F application, as recommended by previous studies. [23].

**Results**

9. It is suggested to revise the results portion to make a clear comparison of different treatments with control treatment.

**Response**: Thank you for the reviewer’s suggestion. We have made appropriate revision.

10. Line 160-161: Make a clear comparison either water and fertilizer caused increased or decreased growth indexes compare to control.

**Response**: comparison of growth parameters with control treatment have been added in revised manuscript. overall, the water and fertilizer coupling treatment increased Pn, Ci, Tr; decreased WUE.” Please see Line 191-194 of the revision.

11. Line 179-180: Again, I suggest to make a clear comparison either water and fertilizer caused increased or decreased growth indexes compare to control.

**Response**: Revised as per suggestion. overall the water and fertilizer coupling treatment increased soluble protein, soluble sugar, SOD,chlorophyll; decreased MDA. Compared with the control, F3W3 promoted the accumulation of soluble protein, while F1W3 and F1W4 decreased the soluble protein content of leaves.” Please see lines 205-208 of the revision.

12. Line 182-183: Same as above.

**Response**: Thank you for the reviewer’s suggestion. “Thus, the treatments with low fertilizer coupled with medium to high water were not conducive to soluble protein accumulation in the blueberry leaves.” changed to “Thus, F1W3 and F1W4 were not conducive to soluble protein accumulation in the blueberry leaves.” Please see lines 208-209of the revision.

13. Line 187-189: Increase of MDA contents indicates plants membrane damage then how increase of MDA content under low/medium fertilizer-high water could be conductive to leaf growth?

**Response**: Thank you for the reviewer’s suggestion. Thank you for pointing this out. According with your advice, we corrected the relevant part in manuscript. Please see line 216-218 of the revision, described it as “Leaf MDA levels were increased in control treatment under missing fertilizer and water application while other treatment combinations have decreased leaf MDA level. Therefore, the medium fertilizer–medium water treatments were most conducive to leaf growth’’.

14. Line 217: Author mentioned that F1W4 represents the lowest membership function value however table 8 represented that CK had lowest membership function value. Please correct it.

**Response**: Thank you for your comments. We have revised it “CK had the lowest average membership function values .”

15. Line 224-225: No appropriate in here, suggested to delete.

**Response**: OK, we deleted it.

16. Please, avoid re-mentioning results repeatedly in the discussion. This was already mentioned in the Results section. What happens with the nutrients and the lack of interaction between the doses of N and P? Why does that happen? Please explain.

**Response**: Thank you for the reviewer’s suggestion, we have made changes in Line 300-309 of the revision.

17 And 18. Line 232-233 and lines 235-237: What is the actual relation of this sentence with the current study?

**Response**: Strawberries are herbs, raspberries are shrubs, and walnuts are trees. It is concluded that blueberries and raspberries are photosynthetically close. It shows that they have similar photosynthetic capacity with strawberries, raspberries and walnuts. We just want to show that the photosynthetic capacity of shrubs lies between trees and herbs; see lines 255-257 of the revision.

19. Line 237-240: Discussion needs to be focused on mechanism rather simply description of verification of results. What kind of strategy is being employed by selected plants and give appropriate mechanism why this happened?

**Response**: It may be due to the fact that under suitable water conditions is beneficial to increase the stomatal conductance, under suitable conditions, adding fertilizers is beneficial to increase the chlorophyll content, or high water and high fertilizer are beneficial to improve the diversity and stability of soil microbial communities, and are beneficial to the soil ecological environment. Or because it helps to reduce the content of MDA in the leaves, thereby promoting the photosynthesis and transpiration of crops. The influence mechanism of water and fertilizer treatment on photosynthesis is relatively complicated, and further research is needed in the future.

20. Line 240-243: This sentence is not clear, please rephrase.

**Response**: Sentence is rephrased for more clarity. see lines 264-269 of the revision.

21. Line 248-250: Author cited a huge number of Chinese Journal reference; it is suggested to reduce this number and add appropriate International Journal reference.

**Response**: Chinese journal references are reduced and replaced with other studies published in some other international journals.

22. Line 290-295: This portion is the simple description of the cluster analysis results and quite similar with L 220-224. Revise this and focus on key points results and discuss with appropriate mechanism.

**Response**: Thank you reviewers for their comments. Respective description is now added in revised version for explanation; see lines 316-329.

23. Line 467: Why gas-exchange parameters such as Pn, Tr, Ci, Gs and WUE were not included in the Membership Function? If author include these parameters calculate accordingly maybe the average membership value and ranking will be changed.

**Response**: We are agreed with reviewer suggestion. This time, leaf enzyme activity and growth indicators were measured in spring, while photosynthetic characters were measured in summer. In Guizhou, China, the spring and summer seasons vary greatly. In order to reduce the seasonal errors, photosynthetic indicators were not taken together to make Membership function analysis. But even if we add photosynthetic indicators, our best combination is still the middle fertilizer treatment, but the ranking of other treatments did change.

**Reviewers 2**

(a) Author’s measures two oxidative stress parameters (MDA and SOD activity) however authors didn’t give any explanation why these two parameters were considered for investigation and their relationship with fertilizer and irrigation doses either in introduction or discussion section.

**Response**: Thank you for the reviewer’s suggestion. I have added relative explanation in introduction; see lines 55-64.

(b) Authors used plenty of common names of different plant species in the discussion section. Authors should use scientific name along with their common names viz., Cucumber (Cucumis sativus).

**Response**: Thank you for the reviewer’s suggestion. Scientific names of all plant species at their 1^st^ occurrence in whole manuscript.

(c) Plenty of type-setting errors were found throughout the MS. Authors should follow the journal guidelines in writing reference in the reference section.

**Response**: References are now written according to journal format and all type-setting errors are removed.
