# Supplementary material for: Effects of water and fertilizer coupling on the physiological characteristics and growth of rabbiteye blueberry
Source: PLoS One. 2021 Jul 6;16(7):e0254013. doi: 10.1371/journal.pone.0254013 (PMC8259967; doi:10.1371/journal.pone.0254013)
Supplement: Attachment — Submitted filename: Response to reviewers.docx [file pone.0254013.s003.docx]

Editorial office Comment: Figure-1 is referred in text but missed

Response: Figure-1 is included now, please see the line-504 after reference section

Reviewer-2 comments

Comment: This is a much-improved version of a paper I have seen before and the authors have addressed all the issues raised. However, I still feel it needs some works before it is publishable.

Response: we are thankful for your nice suggestions to improve the manuscript
Comment: L163-166: I think that this experiment was not conducted in a plastic shed and no pots also used in here. Moreover, author used membership function not integrated growth performance (IGP) in their analysis. Therefore, it is suggested to revise these sentences according to the experimental design.

Response: we are sorry for this error; correction have been made in revised manuscript. Please see the line 165-176.
Comment: L190-191: "and, by extension" -What is the actual meaning of this?

Response: We have removed this typo error in revised file. Line 194
Comment: L256-257: Please mention the scientific name for strawberry and spruce also. Please the whole manuscript to fix such error.

Response: Scientific names are added now: line-264
Comment: L65: Please mention the scientific name of orange in here.

Response: Added in line 272
Comment: L293-294: Sentence is not clear please revise it.

Response: correction have been made; line-301

Comment: L318: Sentence is not clear.

Response: Mentioned sentence is revised now in line-326
Comment: L417: Author should be more careful during writing the names of the authors name in the reference section. For example: in 23 number reference the author’s name will be Mostofa MG not Mostfa MG.

Response: Correction have been made in line 427
